# The effects of computer-based mindfulness training on Self-control and Mindfulness within Ambulatorily assessed network Systems across Health-related domains in a healthy student population (SMASH): study protocol for a randomized controlled trial

**DOI:** 10.1186/s13063-016-1707-4

**Published:** 2016-12-01

**Authors:** Zarah Rowland, Mario Wenzel, Thomas Kubiak

**Affiliations:** Health Psychology, Johannes Gutenberg University Mainz, Binger Straße 14-16, 55122 Mainz, Germany

**Keywords:** Mindfulness, Network approach, Self-control, Randomized controlled trial, Ambulatory assessment

## Abstract

**Background:**

Self-control is an important ability in everyday life, showing associations with health-related outcomes. The aim of the Self-control and Mindfulness within Ambulatorily assessed network Systems across Health-related domains (SMASH) study is twofold: first, the effectiveness of a computer-based mindfulness training will be evaluated in a randomized controlled trial. Second, the SMASH study implements a novel network approach in order to investigate complex temporal interdependencies of self-control networks across several domains.

**Methods:**

The SMASH study is a two-armed, 6-week, non-blinded randomized controlled trial that combines seven weekly laboratory meetings and 40 days of electronic diary assessments with six prompts per day in a healthy undergraduate student population at the Johannes Gutenberg University Mainz, Germany. Participants will be randomly assigned to (1) receive a computer-based mindfulness intervention or (2) to a wait-list control condition. Primary outcomes are self-reported momentary mindfulness and self-control assessed via electronic diaries. Secondary outcomes are habitual mindfulness and habitual self-control. Further measures include self-reported behaviors in specific self-control domains: emotion regulation, alcohol consumption and eating behaviors. The effects of mindfulness training on primary and secondary outcomes are explored using three-level mixed models. Furthermore, networks will be computed with vector autoregressive mixed models to investigate the dynamics at participant and group level. This study was approved by the local ethics committee (reference code 2015_JGU_psychEK_011) and follows the standards laid down in the Declaration of Helsinki (2013).

**Discussion:**

This randomized controlled trial combines an intensive Ambulatory Assessment of 40 consecutive days and seven laboratory meetings. By implementing a novel network approach, underlying processes of self-control within different health domains will be identified. These results will deepen the understanding of self-control performance and will guide to just-in-time individual interventions for several health-related behaviors.

**Trial registration:**

ClinicalTrials.gov, NCT02647801. Registered on 15 December 2015 (registered retrospectively). ﻿﻿

**Electronic supplementary material:**

The online version of this article (doi:10.1186/s13063-016-1707-4) contains supplementary material, which is available to authorized users.

## Background

Self-control is a core human ability that is used to control one’s own daily reactions and behaviors in order to stay in line with rules or self-set goals by regulating and changing inner experiences or situational circumstances [1, 2]. Successful self-control performance is positively associated with mental health outcomes such as affective wellbeing and life satisfaction [3]. Moreover, whereas self-control is positively related to physical health behaviors, such as physical activity and less sedentary behavior [4], it is also negatively associated with a greater obesity risk and a higher Body Mass Index [5] or a greater risk in reporting substance use or binge drinking [6]. Understanding the cognitive-affective processes of self-control is paramount, when it comes to devising and testing interventions and preventive measures to facilitate self-control and to avoid self-control failures that can have a detrimental impact on health behaviors and outcomes.

In recent years, self-control research has increasingly relied on in-situ data collection methods such as electronic diaries or the ambulatory recording of physiological concomitants of self-control (e.g., references [7–9]). These “real-life” assessment approaches, summarized under the umbrella concept of Ambulatory Assessment [10], offer unique advantages to investigate behavioral processes as they unfold in the participants’ daily life and are very well suited to avoid the memory biases that afflict retrospective self-report measures. Ambulatory Assessment allows for tapping into the momentary experiences and the situational context that impact the enactment of self-control in a given real-life situation. Due to these advantages, Ambulatory Assessment strategies have been used to study self-control in a broad range of health-related domains, such as smoking [11], emotion regulation [12] or eating behavior [13] and have led to the identification of a range of predictors in these fields.

Mindfulness-based approaches are a promising candidate for supporting self-control in daily life: mindfulness refers to self-regulatory mechanisms that enable an open-minded, nonjudgemental, and accepting kind of awareness of the present moment [14, 15]. Mindfulness-based interventions have already demonstrated positive effects on health-related behaviors in domains where self-control is essential, as is the case in substance abuse [16] or binge eating [17]. Moreover, a large number of randomized controlled trials are currently being conducted to evaluate the effects of mindfulness-based trainings on psychological wellbeing for a range of healthy and clinical populations, such as medical students, pregnant women or patients with cancer [18–20]. The positive effects of mindfulness can be explained by several mechanisms that may lead to changing behaviors – two amongst them are noticing negative experiences, such as pain sensations or depressogenic thoughts and, by observing them nonjudgementally, redirecting attention to the present moment in order to focus on a current task [21]. This leads to the assumption that training mindfulness could increase awareness of one’s own needs and goals [22], which facilitates effective self-control performance in everyday life domains [23]. Thus, mindfulness could have a positive impact on attention which results in noticing situational cues, inner experiences and goals more easily. The SMASH study implements a mindfulness training to examine the effects of mindfulness on self-control performance.

Since mindfulness should affect self-control enactment, it is also essential to understand how mindfulness training changes regulatory mechanisms which should, in turn, result in improved self-control in the course of an intervention. With the purpose of conceiving the dynamic relationships between processes of mindfulness and self-control, a novel network approach [24] is used to examine underlying self-control processes and the impact of mindfulness as it unfolds across time. This network approach uses multilevel vector autoregressive (VAR) models to model time dynamics between several regulatory components. Time dynamics can then be visualized in an individual or population-network consisting of nodes (variables) and edges (relationships between variables).

Overall, this trial assesses self-control domains in everyday life (following the example of Hofmann et al. [8]) to investigate the effect of a mindfulness training on daily self-control in health-related domains. Further exploratory analyses are conducted with the network approach [24], gaining deeper insights into dynamic relations between underlying processes of self-control.

### Choice of comparator

In order to differentiate between time and true treatment effects of the mindfulness training, a wait-list control condition is included. The control condition does not receive any intervention during the study period but takes part in an Ambulatory Assessment and weekly laboratory sessions to complete self-report questionnaires. Since we expect a positive effect on momentary mindfulness in everyday life for the intervention condition, the wait-list control condition will be offered a comparable mindfulness training after the end of the study.

### Objectives

#### Primary objective

The primary objective of this trial is to determine whether practicing momentary mindfulness (first primary outcome) mediates momentary self-control in daily life (second primary outcome). Of further interest are changes in habitual mindfulness and self-control (secondary outcomes) in the intervention condition compared to a wait-list control condition. Further outcomes are momentary attention control, affect and emotion regulation and key behavioral domains influenced by self-control (eating behavior, sexual behavior, work behavior, interpersonal behaviors, alcohol consumption, spending money).

#### Secondary objective

A novel network approach [24] will be used to model the interplay of momentary self-control with internal and external factors, such as affect, desire strength and positive or negative events, over time. These time-to-time relationships will be analyzed at individual and group level by using multilevel VAR models. Afterwards, different network parameters will be computed exploring overall and local network structure (see “Statistical analysis”). In doing this, (1) underlying processes of self-control enactment become visible, (2) predictors of self-control from a range of different domains will be identified and (3) it is possible to examine mediating processes that convey the effect of mindfulness training on momentary mindfulness, and ultimately on self-control in daily life.

## Methods

**Fig. 1:**
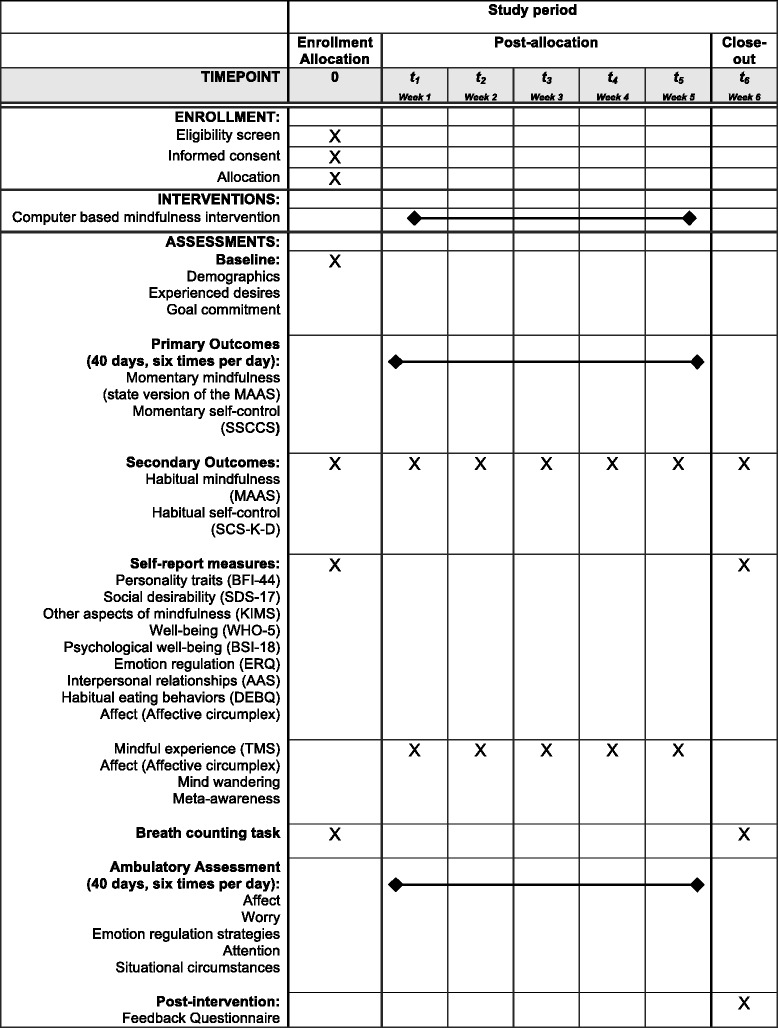
Participant time-line. Both conditions come to all assessment time points and take part in an Ambulatory Assessment. Ambulatory Assessment starts 1 day after the baseline assessment: for 40 consecutive days, signals are distributed six times a day (all items are listed in Additional file 2). *KIMS* Kentucky Inventory of Mindfulness Scale, *BFI-44* Big Five Inventory-44, *MAAS* Mindful Attention Awareness Scale, *SCS-K-D* Self-control Scale-K-D, *SDS-17* Social Desirability Scale-17, *WHO-5* World Health Organization Five Wellbeing Index, *BSI-18* Brief Symptom Inventory, *ERQ* Emotion Regulation Questionnaire, *AAS* Adult Attachment Scale, *DEBQ* Dutch Eating Behavior Questionnaire, *TMS* Toronto Mindfulness Scale. The post-intervention Feedback Questionnaire assesses effort that was expended during Ambulatory Assessment

The SMASH study received approval by the Ethics Committee of the Institute of Psychology in Mainz (reference code 2015_JGU_psychEK_011) and adopts all principles of the Declaration of Helsinki [25]. The SMASH study protocol is reported according to Standard Protocol Items: Recommendations for Interventional Trials 2013 (SPIRIT). A SPIRIT Checklist (Additional file 1) and a SPIRIT figure (Fig. 1) are provided. Protocol modifications will be uploaded to ClinicalTrials.gov.

### Study design and settings

The SMASH study is a randomized, controlled trial that combines an Ambulatory Assessment part of 40 consecutive days and seven weekly laboratory sessions during this period (see Fig. 1). Participants are randomized to either mindfulness training or a wait-list control condition: participants in both conditions attend the weekly laboratory sessions at the Institute of Psychology in Mainz completing self-report measures on habitual self-control and habitual mindfulness. In the intervention, participants additionally engage in intense computer-based mindfulness training.

### Eligibility criteria

Inclusion criteria are (1) the ability to understand and fluently speak German, (2) students (senior students included) aged between 18 and 65 years, (3) sufficient experience to operate a smartphone reliably and (4) having provided informed consent. Exclusion criteria are (1) the diagnosis of a psychiatric disorder and (2) any mental or somatic impairment that precludes the use of a smartphone.

### Intervention

The mindfulness training starts 1 week after the first laboratory meeting. From this time on, participants from the intervention and control conditions take part in different laboratory meetings (separated from each other) to minimize the chance of contamination between conditions. Mindfulness is practiced with a computer-based guided-breathing meditation for five weekly sessions in which one’s own breath has to be counted repetitively from one to nine [26]. A “click” sound occurs if a breath was miscounted guiding attention back to the current moment and the Breath-counting Task [26]. After the first mindfulness training session, participants in the intervention condition are also able to practice mindfulness (breathing meditation or body scan) at home by listening to an audio file via their smartphones. Each episode of ambulatory mindfulness practice is saved in a data file in order to assess how often mindfulness is practiced during the treatment period. After the third training session, participants perform an informal mindfulness task, which is used in mindfulness-based stress-reduction programs [15] to apply mindfulness in everyday life.

Since only healthy participants are enrolled in the SMASH study, we do not expect any harm from the low-level mindfulness intervention. For this reason, there are no criteria for discontinuing or modifying the intervention.

### Primary and secondary outcome measures

#### Momentary mindfulness

The first primary outcome of this trial is momentary mindfulness assessed via electronic diaries six times per day on 40 consecutive days between 10 a.m. and 8 p.m. with three selected items from the state version of the *Mindful Attention Awareness Scale* (MAAS; study 4 from Brown and Ryan [27]). The state version of the MAAS consists of five rephrased items drawn from the original MAAS (Items 3, 8, 10 13 and 14) in order to assess the degree of mindfulness experienced directly in daily life [27]. We chose only three items from the German MAAS [28] (Items 8, 10 and 14) to lower participants’ burden and adapting to the needs of an Ambulatory Assessment. The three items were rephrased according to Brown and Ryan [27].

#### Momentary self-control

The second primary outcome is momentary self-control which is measured via electronic diaries six times per day on 40 consecutive days between 10 a.m. and 8 p.m. with three items from the German version *State Self-control Capacity Scale* (SSCCS) [29]. The SSCCS consists of 25 items and assesses self-control capacity that is momentarily available for self-control execution (Cronbach’s *α* ≥ .93). For the SMASH study, the original SSCCS items 12, 16 and 18 were selected due to their high loadings on the SSCCS: “I can’t absorb any information,” “I want to give up,” “I feel like my willpower is gone.” The selected SSCCS items are complemented with items that were used by Hofmann et al. [7] (see Additional file 2: Table S1 and Table S2).

#### Habitual self-control

One of the secondary outcomes is habitual self-control which is measured with the brief *Self-control Scale* (SCS) consisting of 13 items [2]. The SMASH study uses the German Self-Control Scale-K-D (SCS-K-D) (Cronbach’s *α* = .79 (t1) and *α* = .80 (t2) and a test-retest reliability of *r* = .82) [30]. The SCS-K-D is answered at seven weekly meetings in order to measure changes in habitual self-control.

#### Habitual mindfulness

As a further secondary outcome, habitual mindfulness is assessed with the original version of the MAAS [27], which consists of 15 items. A validated German version of the MAAS is used in this study (Cronbach’s *α* = .83; test-retest reliability *r* = .82) [28]. The self-report of habitual mindfulness is collected at the seven weekly laboratory meetings.

### Additional measures

A range of demographic variables, self-report questionnaires and ambulatorily assessed measures were collected. All self-report and behavioral measures and their assessment time points are shown in Fig. 1 in overview.

#### Demographics

Participants are asked to report their (1) age, (2) gender, (3) highest graduation, (4) engagement in a religion, (5) Yoga practice and (6) substance use (alcohol, cigarettes, marijuana).

#### Self-report questionnaires

The frequency of *experienced desires* in daily life is measured with 15 desire domains [7, 8].

Also *goal commitment* is assessed by asking whether participants have planned to pursue 20 specific goals [7, 8] for the next 6 weeks. Personality traits are assessed with the German version of the *Big Five Inventory-44* (BFI-44) [31, 32], which consists of 42 items and five scales (Cronbach’s *α* = .63 to *α* = .86; test-retest correlation *r* = .55 to *r* = .82) [32]. Furthermore, social desirability is assessed with the *Social Desirability Scale-17* (SDS-17) [33, 34] (Cronbach’s *α* = .72; test-retest correlation *r* = .80) [34] consisting of 16 items.

Complementary to the MAAS, different aspects of mindfulness (observing, acting with awareness, acting without judgement, and describing) are assessed with the 39 items from the German *Kentucky Inventory of Mindfulness Skills* (KIMS) [35, 36] (Cronbach’s *α* = .79 to *α* = .92; test-retest correlation *r* = .78 to *r* = .86) [36]. Additionally, two factors of mindful experience (curiosity and decentering) are measured with the *Toronto Mindfulness Scale* (TMS) [37], which consists of 13 items (Cronbach’s *α* = .95).

Wellbeing is measured with the German version of the *WHO-5 Wellbeing Index* [38] consisting of five items (Cronbach’s *α* = .92 [39]). As another measure for psychological wellbeing, the German version [40] of the *Brief Symptom Inventory-18* [41] is additionally used to assess changes in syndromes of somatization, depression and anxiety with 18 items (Cronbach’s *α* ranges between *α* = .63 and *α* = .93) [40].

Moreover, habitual behaviors in several self-control domains are assessed. The use of two different emotion regulation strategies, reappraisal and suppression, is measured with the German version of the *Emotion Regulation Questionnaire* (ERQ) [42, 43] which consists of 10 items (reappraisal *α* = .76; suppression *α* = .74) [43]. Interpersonal relationships are measured with the German version of the *Adult Attachment Scale* (AAS) [44, 45] consisting of 15 items and the three scales of closeness, trust and fear (*α* = .72 to .79) [45]. The habitual eating styles restrictive eating, emotional eating and external eating are assessed with the *Fragebogen zum Ernährungsverhalten-II* (FEV-II, Cronbach’s *α* = .82 to *α* = .92) [46], the German version of the *Dutch Eating Behavior Questionnaire* (DEBQ) [47]. The FEV-II consists of 33 items.

In order to investigate affective dynamics, the measurement of affect is based on *Russell’s affective circumplex model* [48, 49]. Positive and negative affect are each assessed with four items.

#### Behavioral measures

As a behavioral measure of mindfulness, the *Breath-counting Task* is conducted [26]. During this task, participants have to count their breath for 18 min repetitively from one to nine. Additionally, participants are asked to press a button whenever they caught themselves miscounting their breath (self-caught misc﻿outing)﻿.﻿ Counting accuracy will be analyzed by calculating the percentage of correctly counted breath trials in relation to all trials (self-caught trials, miscounted trials and correctly counted trials). The breath counting is interrupted every 90 s on average in order to present two items, assessing momentary meta-awareness and mind wandering [26].

#### Ambulatory Assessment

Due to conditional branching in the presentation of items during the Ambulatory Assessment, participants answer 31 to 50 items (Additional file 2: Tables S1, S2 and S3) at each signal six times per day regarding (1) positive and negative affect [48, 49], (2) worrying [24, 50], (3) momentary emotion regulation strategies [12], (4) attention (item from previous research in our laboratory) and (5) situational circumstances (items from several Ambulatory Assessment prompts [8, 11, 12, 51]). If a participant indicates a specific desire, additional items appear on the smartphone screen depending on the indicated desire: for example, if a given participant indicates the desire “food”, items are presented that assess specific eating behaviors. Further additional items from the (6) DEBQ [47], (7) the *Situational Motivation Scale* [52], (8) the *Work-related Flow Inventory* [53], (9) the *State Adult Attachment Measure* [54], (10) the *Recovery Experience Questionnaire* [55] and items regarding (11) sexual behavior and (12) spending money are presented in Additional file 2: Tables S2 and S3. A pre-test (*N* = 6) showed that it takes about 2 min (*M* = 1.89; *SD* = 1.84) to complete the presented questionnaires after each signal.

At the time point of study enrollment, there was no method that could model different types of variables in a network. Due to the chosen mode of statistical analysis (network approach) that includes VAR and multilevel regression models, ambulatorily assessed variables all had to be either continuous or binary variables. For this reason, all originally binary variables were changed into continuous variables.

### Procedure

A participant time-line of enrollment, intervention and assessments is presented in Fig. 1. During a first laboratory session, a research assistant conducts a screening interview to check for inclusion and exclusion criteria. Eligible participants are invited to give their written consent for study participation (see Additional file 3). Afterwards, allocation takes place. Participants are free to withdraw their consent to participate anytime without any consequences.

#### Laboratory sessions

During the first laboratory session, all participants complete self-report questionnaires and a Breath-counting Task [26] which is conducted on computer. One week after the first laboratory meeting, both intervention and control condition participants come to five weekly laboratory meetings completing questionnaires regarding self-reported dispositional mindfulness and self-control. A maximum of three participants can take part in a laboratory meeting at once. From the second laboratory meeting on, only the intervention condition starts practicing mindfulness with a computer-based guided-breathing meditation for 12 min at five weekly appointments. At the seventh and final laboratory meeting, a post-intervention measurement takes place. During the final measurement, questionnaires that were completed within the first laboratory meeting are answered again. Also the Breath-counting Task [26] is conducted by both control and intervention conditions comparing counting accuracy between them as well as before and after mindfulness training. The seven laboratory meetings are conducted on computers with the operating system OS X El Capitan, Version 10.11 and a screen resolution of 1920 × 1080. The software Inquisit 4 (Millisecond Software, 2014) is used to present the questionnaires on the screen.

#### Ambulatory Assessment protocol

A day after the first laboratory session, the Ambulatory Assessment begins (day 1 till day 40). Each day, signals are randomly distributed six times a day via smartphones throughout a defined timeframe of 10 h. Signals can occur between 10 a.m. and 8 p.m. This time span is divided into blocks of 2 h [56], defining for each block that two consecutive signals could be at least 45 min apart. Throughout a timeframe of 5 min after a signal, participants can start, reject or postpone their answer for 15 min. Participants are asked to complete short questionnaires which assess experiences at the present moment or within the last 30 min [7].

Moto E (second generation) smartphones with the latest Android Lollipop version 5.1 are handed to participants in case they do not own an Android-based smartphone. Ambulatory Assessment is run via the application movisensXS, version 0.8.4203 (movisens GmbH, Karlsruhe, Germany), which has to be downloaded and installed either on the personal or the received smartphone. This app schedules the beeping signals, presents the questionnaires on the smartphone and saves collected data by uploading it to a secured server.

### Sample size

The sample size was calculated with a power calculation formula for multilevel models [57] to detect group differences in state and trait mindfulness between individuals. Based on previous research in our laboratory, we assume an unexplained intraclass correlation (ICC) of 0.25 and want to achieve a power of 1 − *β* = .95 and set an alpha level *α* = .05 for a two-sided test to detect a medium difference of Cohens’s *d* = .33 between conditions. The expected medium effect size is based on a meta-analysis (medium effect size Hedge’s *g* = .33) comparing mindfulness-based therapy treatment with other active treatments [58] – since the wait-list control condition participants take part in an Ambulatory Assessment and come to weekly laboratory meetings during its waiting period, we considered the wait-list control condition as an active treatment condition as a conservative estimate for our power calculations rather than an inactive waiting condition. Thus, this leads to a sample size of *N* = 120 to detect medium group differences between individuals. Since prior Ambulatory Assessments in our department showed attrition rates of approximately 10%, the SMASH study aims at a sample size of *N* = 134 to achieve a final total *N* = 120 (control condition *n* = 60; mindfulness intervention *n* = 60).

### Recruitment

Participants are to be recruited from the undergraduate student population of psychology on campus at the Institute of Psychology, Johannes Gutenberg University Mainz, Germany. Flyers are pinned on blackboards and the study is advertised during lectures, seminars and via social networks. Recruitment and enrollment started in October 2015 and will take place continually during the execution of the trial till the expected sample size is achieved (December 2016).

As a means of compensation, the student participants receive course credits. Additionally, all participants who complete more than 80% of the daily questionnaires participate in a raffle of two 100 EUR vouchers for an online shopping website.

### Allocation and blinding

Since we want to reach a sample size of 134 participants, we randomized the total number of participants preliminary to the trial. We used the random allocation rule as a restricted randomization procedure [59] to generate a sequence that randomly orders 67 intervention and 67 control group assignments. Based on this random allocation sequence, consecutively enrolled participants are sequentially assigned to either the control or the intervention condition so that a balanced group allocation should arise at the end of the trial. However, attrition could result in imbalanced group sizes, regardless of the chosen randomization technique. Research assistants who are in personal contact with participants did not prepare the coding list. Preliminary allocation to a condition will not be changed. Due to the wait-list control design, blinding is not possible.

### Statistical analysis

Data analysis will be conducted with Stata 13 (College Station, TX, USA: StataCorp LP) and R (R Development Core Team, Vienna, Austria, 2008) [60]. Analyses include descriptive statistics, mixed models and multilevel VAR models [24]. Missing observations will not be imported for data analysis.

### Primary and secondary outcome analysis

First, the effect of mindfulness training on momentary mindfulness and self-control will be explored. Since Ambulatory Assessment data have a hierarchical structure, where several observations are nested within each day and days are nested within individuals, we use three-level multivariate mixed models including random effects for each level-1 variable (daily observations) allowing for within-day (level 2) and within-person (level 3) variations. In this way, time-varying momentary mindfulness and self-control on level 1 (daily observations) will be predicted by day (level 2) and group (level 3), controlling for age, gender, social desirability and other personality traits at level 3. Significant main effects of the variables day and group would indicate group differences and a change of momentary mindfulness and self-control across time. Furthermore, significant two-way interactions between the continuous variable day (level 2) and the categorical variable group (level 3) would indicate an effect of mindfulness training on momentary mindfulness and self-control relative to the control condition in everyday life.

The secondary outcomes habitual mindfulness and self-control are examined using mixed models. Two-way interactions between laboratory meetings (seven meetings) and group (mindfulness versus control) predicting habitual mindfulness and self-control would indicate differences between groups from one meeting to another. Further additional momentary and habitual measures will be examined in a similar manner.

### Additional analyses

#### Self-control networks

**Fig. 2:**
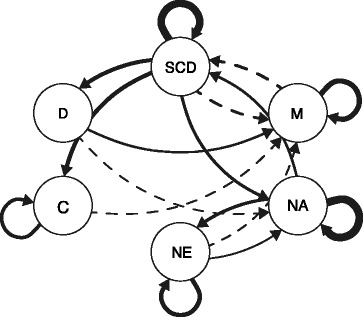
Time-to-time dynamics between several variables at baseline (day 1 till day 6). Variables: SCD = self-control depletion; M = mindfulness; NA = negative affect; NE = negative event; C = conflict; D = desire strength. Solid arrows represent positive relations and dashed arrows represent negative relations. The thicker the line the higher is the fixed-effect coefficient. In this case, only coefficients reaching significance are presented (*p* < .05)

Dynamic relations between self-control variables will be analyzed and visualized with the network approach [24] for the baseline (week 1) and post-intervention period (week 6) as well as for other timeframes. For computing estimates of the multilevel VAR models, the R package mlVAR will be used [61]. Networks will be constructed out of selected ambulatorily assessed variables (e.g., self-control depletion, mindfulness, negative affect, negative event, conflict and desire strength). First, multilevel VAR models are modelled for each network variable – every single network variable is regressed to a lagged version of itself and all other independent lagged variables (values that were measured at a previous time point). These multilevel VAR models contain fixed and random effects where the fixed effects are the average population effects of the lagged variables on the dependent variable (group level) and the random effects the participant’s deviations of the average population effect (individual level). Then, the fixed-effects coefficients of all modelled multilevel VAR models are used to visualize directed relationships between all network variables. The self-control population network containing average connection strength between variables at group level will be visualized with the qgraph R package [62]. In accordance with Bringmann et al. [24], the False Discovery Rate [63] will be controlled at 5% in order to control for multiple testing. An exemplary network is presented in Fig. 2 showing time-to-time relations (edges) between network variables (nodes). For a more detailed description, see Bringmann et al. [24]. This procedure will also be applied to construct population networks for the control as well as for the mindfulness condition in specific health domains like alcohol consumption, emotion regulation and eating behavior.

#### Individual self-control networks

By using a subject’s individual random effects with the fixed-effect coefficients of the multilevel VAR models, networks can be visualized that show only temporal dynamics of a specific subject. In this way, individual self-control networks can be visualized [24].

#### Effects of mindfulness on the self-control network

In order to investigate the effect of mindfulness on the interconnections of the self-control network, a treatment variable (mindfulness versus control) and a time period variable (pre versus post intervention) will be added to the multilevel VAR models of the population network [24]. Differences between mindfulness and control conditions can be examined by looking at single links between two specific variables within the network [24]. If there is a change across time at a specific link between a lagged independent and a dependent variable that differs between conditions, there should be a significant three-way interaction between the variables treatment, time period and the independent lagged variable on the dependent variable.

#### Network structure

To assess local and overall network structures and their changes, several network parameters, such as network density (overall connectivity) and centrality (In-strength, Out-strength, Closeness and Betweenness), will be analyzed for individual and population networks [64]. Density indicates the overall connection strength between all nodes (variables) and is calculated by averaging over the absolute values of all regression coefficients. High density represents strong interconnections between the network variables which means they have high impact on each other from one time point to the next. Different centrality indices [65] will be computed for all network variables to explore the importance within the network of each of them. Those indices indicate how much information a specific node exchanges with other nodes or how close it lies to the others (for a detailed description see Bringmann et al. [24, 64]).

### Data storage and monitoring

All data is pseudonymized – thus, all individuals participating are rendered unidentifiable. Moreover, ambulatory data is encrypted by movisensXS and will be saved on a secured server of the Host Europe GmbH in Cologne, Germany (ISO 27001 certified). All datasets that are saved on this server will be password-protected and only project principal investigators will have access to them. Since the SMASH study conducts a low-level mindfulness training, a Data monitoring Committee is not established. Data monitoring is the responsibility of the first author. Adverse events or other unintended effects of the trial are not expected. For this reason, interim analysis and stopping guidelines are not provided.

## Discussion

The SMASH study investigates the effects of computer-based mindfulness training on daily mindfulness and self-control, which is a promising way of supporting self-control performance. Furthermore, the novel network approach by Bringmann et al. [24] will provide new evidence concerning general self-control in daily life that is manifested in behavior and experiences within several self-control domains. Dynamic changes of internal states, external cues and self-control performance will be investigated before and after mindfulness training. The network approach allows identifying underlying processes of self-control within different self-control domains and also allows identifying central components of group and even individual networks. This is a promising way to gain a deeper understanding of self-control performance and allows an implementation of just-in-time individual interventions [66] for health-related behaviors such as smoking, eating or emotion regulation.

### Trial status and dissemination

The recruitment began in September 2015. We expect the last participant to finish in December 2016. Dissemination of results will be realized through publications in peer-reviewed journals and conference presentations.

## Additional files

Additional file 1: SPIRIT C﻿hecklist. (PDF 76 kb)
Additional file 2: 
**Table S1.** Ambulatorily assessed items [7, 11, 12, 24, 26, 28, 29, 48–50, 52]. **Table S2.** Additional items if a desire is indicated [7, 8]. **Table S3.** Additional ambulatorily assessed items for specific desires [11]. (PDF 156 kb)
Additional file 3: Informed consent form. (PDF 40 kb)

## Abbreviations

AAS: Adult Attachment Scale
BFI-44: Big Five Inventory-44
BSI-18: Brief Symptom Inventory-18
DEBQ: Dutch Eating Behavior Questionnaire
ERQ: Emotion Regulation Questionnaire
FEV-II: Fragebogen zum Ernährungsverhalten-II
ICC: Intraclass correlation
KIMS: Kentucky Inventory of Mindfulness Skills
MAAS: Mindful Attention Awareness Scale
SCS-K-D: Self-control Scale-K-D
SDS-17: Social Desirability Scale-17
SMASH: Self-control and Mindfulness within Ambulatorily assessed network Systems across Health-related domains
SPIRIT: Standard Protocol Items: Recommendations for Interventional Trials
SSCCS: State Self-control Capacity Scale
TMS: Toronto Mindfulness Scale
VAR: Vector autoregressive
WHO-5: World Health Organization Five Wellbeing Index
