# Supplementary material for: The effects of computer-based mindfulness training on Self-control and Mindfulness within Ambulatorily assessed network Systems across Health-related domains in a healthy student population (SMASH): study protocol for a randomized controlled trial
Source: Trials. 2016 Dec 1;17:570. doi: 10.1186/s13063-016-1707-4 (PMC5134121; doi:10.1186/s13063-016-1707-4)
Supplement: Additional file 2: — Table S1. Ambulatorily assessed items [7, 11, 12, 24, 26, 28, 29, 48–50, 52]. Table S2. Additional items if a desire is indicated [7, 8]. Table S3. Additional ambulatorily assessed items for specific desires [11]. (PDF 156 kb) [file 13063_2016_1707_MOESM2_ESM.pdf]

Table S1

Ambulatorily assessed items

Variable	Items	Options
**State self-control capacity** [a]	1) I can't absorb any information.<br>2) I want to give up.<br>3) I feel like my willpower is gone.	1 (not true) – 7 (very true)
**Affect** [b]	1) happy<br>2) excited<br>3) relaxed<br>4) satisfied<br>5) angry<br>6) anxious<br>7) depressed<br>8) sad<br>9) worried [c]	Slider from 1 (not at all) - 100 (very much)
**Desire** [d]	Do you experience a desire right now or have you been experiencing a desire within the last 30 minutes?	0 (no desire at all) – 7 (very strong)
**Social situation** [e]	Right now, I am...	1) alone<br>2) with friends/ acquaintances<br>3) with family<br>4) with coworkers<br>5) with unknown people<br>6) with my girl/ boyfriend
**Social stress** [f]	Within the last 30 minutes, to what degree have you been wishing to be alone rather than being with others?	1 (not at all) - 7 (very much)
**Location** [d, e]	Right now, I am...	1) at home<br>2) in a bar<br>3) in a restaurant<br>4) at work<br>5) at a friend's house<br>6) in a public building<br>7) outside/ in nature

Variable	Items	Options
		8) in a vehicle<br>9) at university
**Alcohol intoxication** [d]	Right now, to what degree are you under the influence of alcohol?	1 (not at all) – 5 (very much)
**Situational exhaustion**	The current situation is exhaustive.	0 (not at all) – 7 (very much)
**Negative event** [g]	Think about the most negative event since the last signal, how intense has it been?	Slider from 1 (no negative event) – 100 (very negative event)
**Positive event** [g]	Think about the most positive event since the last signal, how intense has it been?	Slider from 1 (no positive event) – 100 (very positive event)
**Activity type** [e]	What kind of activity have you been doing for the last 30 minutes?	1) work<br>2) leisure time<br>3) interactivities<br>4) between activities
Type of work	Which kind of work?	1) job<br>2) school/ university<br>3) house/ personal
Type of leisure time	Which leisure type?	1) hobbies<br>2) media<br>3) sports/ exercise<br>4) hanging out<br>5) waiting<br>6) doing nothing
Type of interaction	Which kind of interactivity?	1) socializing<br>2) for work<br>3) household<br>4) arguing
**Attention**	For the last 30 minutes I have had problems concentrating.	1 (not at all) – 6 (very much)
**Meta-awareness** [h]	How aware were you of where your attention has been for the last 30 minutes?	1 (not at all) – 6 (very much)
**Mindfulness** [i]	1) I rush through activities without being really attentive to them.<br>2) I do jobs or tasks automatically, without being aware of what I'm doing.	0 (not at all) – 6 (very much)

Variable	Items	Options
	3) I find myself doing things without paying attention.	
**Emotion regulation** [g]	Since the last beep, I have...	1 (not at all) – 7 (almost all the time)
Reappraisal	… viewed the cause of my feelings from a different perspective.	
Suppression	… suppressed the expression of my feelings.	
Rumination	... brooded about something in the past or future.	
Distraction	… distracted my attention away from my feelings.	
Social support	... talked about my feelings with others.	

Note. All items presented in this table are answered at every single signal six times a day for 40 consecutive days. [a] three SSCCS items [29]; **[b]** based on Russel's affective circumplex model [48,49]; [c] additional item [24,50]; [d] based on Hofmann et al. [7]; [e] based on Shiffman et al. [11]; [f] based on van Winkel et al. [52]; [g] Koval et al. [12]; [h] based on Levinson et al. [26]; [i] items from the MAAS [28].

Table S2

Additional items if a desire is indicated

Variable	Items	Options
**Desire domain**	What kind of desire has it been?	1) food<br>2) nonalcoholic drinks<br>3) alcohol<br>4) coffee<br>5) tobacco<br>6) other substances<br>7) sex<br>8) media<br>9) spending<br>10) work<br>11) social<br>12) leisure<br>13) sleep<br>14) hygiene-related<br>15) other
**Desire duration**	How long have you been experiencing this desire?	1) 0-5 min<br>2) 6-10 min<br>3) 11-15 min<br>4) 16-20 min<br>5) 21-30<br>6) 31-60 min<br>7) 1-2 hr<br>8) 2-3 hr<br>9) 3-5 hr<br>10) > 5 hr
**Conflict**	In case you were experiencing a desire, how much has it been conflicting with a personal goal?	0 (no conflict at all)-4 (very strong conflict)
**Goal**	What kind of goal has it been?	20 options

Variable	Items	Options
**Goal importance**	How important has it been to you to reach this goal?	0 (not important at all)-4 (very important)
**Motivation** [a]	I have had set this goal...	1(not at all) - 7 (very much)
Intrinsic	… because I think it is interesting and fun.	
External regulation	… because it is something I have to do.	
Amotivation	… but I don't know; I don't see what this brings me.	
Identified regulation	… because I think that this is good for me.	
**Resistance**	To which extent have you tried to resist your desire?	0 (not at all) -4 (very much)
**Successful resistance**	How successfully could you resist the desire?	0 (not at all) – 4 (very successful)
**Model for desire resistance**	Which people have successfully resisted the desire you were experiencing?	1) No one<br>2) closely related person<br>3) unknown people<br>4) people in media
**Desire enactment**	To which extent have you enacted on your desire?	0 (not at all) - 4 (very much)
**Model for desire enactment**	Which people have enacted your experienced desire?	1) No one<br>2) closely related person<br>3) unknown people<br>4) people in media
**Another desire**	Have you been experiencing another desire within the last 30 minutes?	0 (no desire at all) – 7 (very strong)
	What kind of desire?	15 desire domains

Note. Items presented in this table will only be presented to participants, if a desire has been experienced. [a] rewritten items from the Situational Motivation Scale; all other items are based on Hofmann et a. [7,8].

Table S3

Additional ambulatorily assessed items for specific desires

Variable	Items	Scale options
**Eating behavior** [a]	How would you describe your eating behavior since the last signal?	1 (not at all) – 5 (very much)
Sate restrictive eating behavior	1) I deliberately ate less (or ate nothing) in order not to become heavier.	
State emotional eating behavior	2) I had a desire to eat when I felt depressed, discouraged or lonely.	
State external eating behavior	3) I had a desire to eat when I saw or smelled something delicious.	
**Sex behavior** [b]	How would you describe your desire for sex since the last signal?	1 (not at all) – 5 (very much)
Restrictive sex behavior	1) I tried not to think about sex.	
Emotional sex behavior	2) I had a desire to have sex when I felt lonely or bored.	
External sex behavior	3) I had a desire to have sex when I saw sexual cues.	
**Flow** [c]	How would you describe your working experiences since the last signal?	1 (not at all) – 7 (totally)
Absorption	1) When I am working, I forget everything else around me.	
Work enjoyment	2) I did my work with a lot of enjoyment.	
Intrinsic Work Motivation	3) I get my motivation from the work itself, and not from the reward for it.	
**Attachment** [d]	To which degree have following statements applied to you for the last 30 minutes?	1 (not at all) -7 (extremely)
Secure	1) I feel loved.	

**Variable**	**Items**	**Scale options**
Ambivalent	2) I feel a strong need to be unconditionally loved right now.	
Insecure	3) If someone tried to get too close to me, I would try to keep my distance.	
**Recovery experiences** [e]	How have you been experiencing your leisure time for the last 30 minutes?	1 (not at all) – 5 (very much)
Psychological detachment	1) I don't think about work at all.	
Relaxation	2) I do relaxing things.	
Mastery	3) I do things that challenge me.	
**Amount of spent money**	How much money have you been willing to spend within the last 30 minutes?	Free input
**Availability** [f]	How easily available have the desired things been within the last 30 minutes?	0(easily available) – 4(available with difficulties)

Note. If a specific desire is experienced, items listed in this table will be answered additionally. Additional items for following desires: [a] food (three items from the Dutch Eating Behavior Questionnaire (DEBQ), German: Fragebogen zum Ernährungsverhalten); [b] sex (three rewritten items from DEBQ); [c] work (three items from the Work-related Flow Inventory (WOLF); [d] social contact (three items from the State Adult Attachment Measure (SAAM); [e] leisure time (three items from the Recovery Experience Questionnaire); [f] food, alcohol, coffee, cigarettes, nonalcoholic drinks or other substances (availability item based on Shiffmann et al. [11]).
