# Supplementary material for: The effects of computer-based mindfulness training on Self-control and Mindfulness within Ambulatorily assessed network Systems across Health-related domains in a healthy student population (SMASH): study protocol for a randomized controlled trial
Source: Trials. 2016 Dec 1;17:570. doi: 10.1186/s13063-016-1707-4 (PMC5134121; doi:10.1186/s13063-016-1707-4)
Supplement: Additional file 3: — Informed consent form. (PDF 40 kb) [file 13063_2016_1707_MOESM3_ESM.pdf]

S.M.A.S.H.-Studie
Einwilligungserklärung

Studiendurchführung: Zarah Rowland
zarowlan@uni-mainz.de
Abteilung Gesundheitspsychologie
Psychologisches Institut
Johannes Gutenberg-Universität Mainz

Ich erkläre mich bereit an der o. g. Studie freiwillig teilzunehmen. Ich bin in einem persönlichen Gespräch ausführlich und verständlich über Ziele, Bedeutung und Zweck der Studie aufgeklärt worden. Ich hatte die Gelegenheit zu einem Beratungsgespräch. Alle meine Fragen wurden zufriedenstellend beantwortet, ich kann jederzeit neue Fragen stellen. Ich habe darüber hinaus die Probandeninformation gelesen und verstanden.
Ich hatte ausreichend Zeit, mich zu entscheiden. Mir ist bekannt, dass ich jederzeit und ohne Angabe von Gründen meine Einwilligung zur Teilnahme an der Studie zurückziehen kann (mündlich oder schriftlich), ohne dass mir daraus Nachteile entstehen.

Ich habe verstanden und bin damit einverstanden, dass meine studienbezogenen Daten zunächst pseudonymisiert (d.h. kodiert ohne Angabe von Namen, Anschrift, Initialen oder Ähnliches) erhoben, auf Datenträgern gespeichert und ausgewertet werden.
Die Weitergabe an Dritte einschließlich Publikation erfolgt ausschließlich in anonymer Form, d.h. kann nicht meiner Person zugeordnet werden.
Für den Fall, dass ich die Studienteilnahme widerrufe, werden meine bereits erhobenen personenbeziehbaren Daten umgehend gelöscht.

Zudem wurde ich darüber informiert, dass es movisens GmbH, Karlsruhe, Deutschland gestattet ist, die mit dem Smartphone gesammelten Daten zu verarbeiten und auf der Datenbank der Host Europe GmbH in Köln, Deutschland zu speichern bis die Löschung der Daten von den Studienleitenden angefordert wird. Die Daten werden anonym gesammelt und gespeichert. Die Identität der ProbandInnen ist movisens unbekannt. Der vorliegenden Studie ist nicht gestattet Daten von ProbandInnen mit dem Smartphone zu erheben, die die Identität der ProbandInnen aufdecken könnte.

Ein Exemplar der Studieninformation und Einwilligungserklärung habe ich erhalten, gelesen und verstanden.

Ort, Datum, Unterschrift Proband

Ich habe das Aufklärungsgespräch geführt und die Einwilligung des Teilnehmers eingeholt.

Ort, Datum, Name der Versuchsleitung in Druckbuchstaben und Unterschrift
